# A global perspective on adaptive radiation: advances, issues, and future directions

**DOI:** 10.1093/evolinnean/kzaf009

**Published:** 2025-05-15

**Authors:** James Stroud, Julia J. Day, María del Rosario Castañeda, Christopher H. Martin

**Affiliations:** 1 School of Biological Sciences, Georgia Institute of Technology, Atlanta, GA 30030, United States; 2 Department of Genetics, Evolution and Environment, Gower Street, University College London, WC1E 6BT, United Kingdom; 3 Departamento de Ciencias Biológicas, Biotecnología y Bioprocesos, Universidad Icesi, Cali, Valle del Cauca, Colombia; 4 Department of Integrative Biology, University of California, Berkeley, Berkeley, CA 94720-3140, United States; 5 Museum of Vertebrate Zoology, University of California, Berkeley, Berkeley, CA 94720, United States

Adaptive radiation—the evolutionary divergence of members of a single phylogenetic lineage into a variety of different adaptive forms (Futuyma 1998)—is widely considered responsible for generating much of Earth’s remarkable diversity (Simpson 1953, Gillespie 2024). Although a unified definition of ‘adaptive radiation’ has attracted much debate in the literature (Olson and Arroyo-Santos 2009, Glor 2010, Givnish 2015), the general process is, and has been, of great interest to evolutionary biologists throughout the history of the field.

This special issue, ‘A global perspective on adaptive radiation’, is intended to bring together new insights on the theory, processes, genetic mechanisms, and ecological contexts driving adaptive radiation across a wide variety of taxa and geographical regions. Contributions range from novel conceptual insights surrounding the evolutionary dynamics of adaptive radiation (Grant and Grant 2024, Schluter 2024, Stoy *et al*. 2024, Turner 2024) to empirical studies and reviews on vertebrates, including fishes (Chan *et al*. 2024, Conith *et al*. 2024, Fenton *et al*. 2024, Nicholas and López-Fernández 2024, Peart *et al*. 2024), early tetrapods (Berks *et al*. 2025), lizards (Salazar *et al*. 2025, Singhal 2025), birds (Grant and Grant 2024), bats (Santana *et al*. 2024), invertebrates (Layfield *et al*. 2024, Van Bocxlaer *et al*. 2024, Yang *et al*. 2024), and plants (dos Santos *et al*. 2024, Zapata *et al*. 2024).

Two contributions in this special issue (Grant and Grant 2024, Schluter 2024) are from Darwin–Wallace medal winners, one of the Linnean Society of London’s highest honours and awarded for ‘major advances in evolutionary biology’. Peter and Rosemary Grant (2009 awardees) use insights from their career-long study of Darwin’s finches in the Galápagos to highlight the role of hybridization in adaptive radiation (Grant and Grant 2024). Dolph Schluter (2014 awardee) provides similarly valuable conceptual insights, discussing issues surrounding a perennial problem in adaptive radiation research: why has there been mixed success in linking micro- and macroevolution?

Across all contributions, we have identified five emerging themes in adaptive radiation research. Below we introduce these themes and discuss the roles that each contribution plays in advancing our understanding of adaptive radiation.

## MAJOR EMERGING THEMES IN THIS ISSUE

### Bridging the process–pattern divide

A classic problem in adaptive radiation research has been the extent to which microevolutionary processes predict macroevolutionary patterns, and vice versa (Schluter 2024). Bridging this divide is proving a difficult problem to solve in both adaptive radiation specifically, and evolutionary biology more broadly (Rolland *et al*. 2023, Stroud and Ratcliff 2025). For the most part, the stages of adaptive radiation in which microevolutionary processes drive diversification typically happened in the deep past and so cannot be directly observed (Gillespie *et al*. 2020, Stroud and Losos 2020). Instead, these historical processes must typically be inferred from present-day patterns of diversity and trait distributions. Schluter (2024) discusses some issues with bridging this process–pattern divide between micro- and macroevolution, focusing on three primary issues: (i) the relative roles of genetic variation versus natural selection in driving patterns of species divergence, (ii) why the rate of evolution of reproductive isolation surprisingly fails to explain species diversification rates, and (iii) the extent to which present-day patterns of natural selection among populations can be predicted from the distribution of species’ phenotypes in a clade. From this, Schluter proposes that a major bridge linking micro- and macroevolution—one that would increase our understanding of adaptive radiation—would be to estimate adaptive landscapes from first principles. This approach involves constructing theoretical models of fitness (or performance) landscapes based on fundamental biological and physical principles, rather than solely from observed data as is most commonly employed in contemporary studies (Martin and Wainwright 2013, Schluter *et al*. 2021, Beausoleil *et al*. 2023, Stroud *et al*. 2023, 2024b). Such models would allow one to predict the fitness of trait combinations, or more complex phenotypes, that do not currently exist in nature and therefore cannot be directly observed or studied (i.e. those that fall in unoccupied regions of the adaptive landscape). For example, theoretical performance surfaces have already been predicted from biomechanical principles in a variety of taxa (Raup 1966, Tseng 2013, Stayton 2019, Olsson *et al*. 2020, Holzman *et al*. 2022, Simon and Moen 2023). However, while these surfaces can reveal whether certain biomechanical interactions constrain macroevolutionary trajectories, they still lack the context-dependence of ecological interactions in nature and a connection to microevolutionary processes. Extending this approach to connect empirical fitness data will provide exceptional insights into how selection drives the correlated evolution of both form and function.

Alternatively, another fruitful approach to bridge this process–pattern divide in adaptive radiation research is to identify young groups that may still be in the process of radiating (Gillespie *et al*. 2020, Stroud and Losos 2020). Such groups provide an exceptional opportunity to study diversification dynamics in real-time. The long-term evolutionary field study of Darwin’s finch radiation in the Galápagos is a classic example of this approach (Grant and Grant 2014). Drawing from 40 years of research and combining detailed field observations with genomic data, Peter and Rosemary Grant’s contribution to this special issue formalizes their newest perspective on adaptive radiation: the ‘competition–selection–hybridization’ model (Grant and Grant 2024). In this new framework, the Grants suggest that hybridization between sympatric taxa can be a powerful generator of species diversity, re-casting the traditional ‘allopatry-then-sympatry’ model whereby taxa differentiate first in allopatry and then natural selection drives further divergence upon secondary contact in sympatry (Schluter 2000). Of course, the recognition of hybridization playing an important role in adaptive radiations is not new (e.g. Seehausen 2004, 2013, Mallet 2009, Schenk 2021), and widespread hybridization has been uncovered in a wide range of taxa using genomics techniques (Brawand *et al*. 2014, Meier *et al*. 2017, 2023, Kozak *et al*. 2021, De-Kayne *et al*. 2022, Patton *et al*. 2022, DeBaun *et al*. 2023, Wogan *et al*. 2023). While many of these groups are considered ‘young’ and so gene flow may be expected, recent studies have begun to uncover evidence for similar ancestral patterns in comparatively older radiations (DeBaun *et al*. 2023, Wogan *et al*. 2023).

Clearly, understanding the role of hybridization represents a major objective for adaptive radiation research (Seehausen 2004, Kagawa and Seehausen 2020, Anderson and Matute 2025). Several important questions appear outstanding: Is hybridization more common in some taxa than others? Are hybridization events equally likely across all lineages within a radiation? Do groups with high levels of hybridization exhibit greater adaptive diversity than those in which hybridization appears rare? In what situations does hybridization ‘hinder’ adaptive radiation by collapsing incipient speciation events before reproductive isolation is complete? While many of the mechanistic questions will probably be answered by applying increasingly sophisticated genetic techniques to well-studied groups, understudied radiations—many of which are highlighted in this special issue—are a valuable, if not necessary, opportunity to test the ubiquity of the hybridization model more broadly.

### Drivers of adaptive radiation

Access to ecological opportunity is widely considered an important trigger for adaptive radiation (Schluter 2000, Wellborn and Langerhans 2015, Stroud and Losos 2016, Fenton *et al*. 2024). A classic example is the geographical colonization of new environments, such as islands or lakes, that may provide access to many new or unoccupied niches that a lineage can radiate to fill (Simpson 1953, Gillespie 2004, Seehausen 2006, Stroud and Losos 2016, Herrmann*et al*. 2021). For example, in this issue, Fenton *et al*. (2024) find that larger lake ecosystems are associated with higher genetic and phenotypic diversity and a higher probability of trophic specialization in Arctic charr (*Salvelinus alpinus*) in Scotland. Similarly, Berks *et al*. (2025) demonstrate that the ecological transition of early tetrapods to herbivory triggered adaptive radiation in the group. Despite these tetrapods transitioning from aquatic to terrestrial environments, it was only after herbivory evolved that the group underwent an extensive adaptive radiation into a variety of feeding specialists exhibiting a range of jaw morphologies.

Mountains can also provide similar environmental opportunities for adaptive diversification. The emergence of the Andean mountains, for example, provided a suite of new cool, high-elevation habitats that many South American tropical low-elevation taxa could radiate into (Monasterio and Sarmiento 1991, Hughes and Eastwood 2006, Ceccarelli *et al*. 2016, Pouchon *et al*. 2018, 2021, Esquerré *et al*. 2019, Guevara-Andino *et al*. 2024). In this issue, Salazar *et al*. (2025) demonstrate that the Andes are also an integral environmental feature driving the adaptive radiation of *Anolis* lizards (anoles). Across the Andes, different anole species that occupy similar elevational bands have evolved similar thermal physiologies. For example, high-elevation species have independently evolved greater cold tolerance compared to their lowland counterparts. Local physiological adaptation to discrete elevational bands in tropical mountains probably contributes to species diversification by minimizing the extent to which species can disperse, therefore limiting gene flow across elevational gradients (Janzen 1967, Polato *et al*. 2018, Linck *et al*. 2020, 2021).

Similar to the emergence of new environments, phenotypic innovations—often called ‘key’ innovations in adaptive radiation research (Schluter 2000, Miller *et al*. 2023)—can also promote diversification. Here, Stoy *et al*. (2024) discuss the evolution of complex multicellularity as a key innovation driving adaptive radiations across major taxonomic groups including animals, plants, fungi, red algae, and brown algae. By analysing three key mechanisms—the evolution of multicellularity and novel functional innovations, the changes in evolutionary dynamics caused by reduced effective population size, and evolutionary priority effects—Stoy *et al*. argue that while broad-scale evolutionary processes may be predictable, specific diversification pathways remain unpredictable.

Two studies of cichlid fishes in this issue also investigate the role of innovations in adaptive radiation. Conith *et al*. (2024) examine the genetic basis of a functionally critical skull bone, the parasphenoid, in Lake Malawi cichlids and demonstrate how small genetic changes in critical morphological traits can have large impacts on ecological function, facilitating adaptive radiation through shifts in trophic specialization. The second set of jaws of cichlids (pharyngeal jaws) have been suggested to be a ‘key innovation’ in their evolution (Liem 1973). In this issue, Nicholas and López-Fernández (2024) examine macroevolutionary trends of this entire trait complex in Neotropical cichlids by harnessing microcomputed tomography (μCT) scanning, a cutting-edge 3D imaging technique. In contrast to some lacustrine cichlid radiations (e.g. Ronco *et al*. 2021), they do not find an early burst ‘adaptive’ signature in this continental radiation, highlighting the nuances of examining multiple trait dimensions across lineages.

The interplay between extrinsic factors, such as the environment, and intrinsic factors, such as morphology and development, can also drive adaptive radiation. Santana *et al*. (2024) discuss this perspective in Neotropical leaf-nosed bats (Phyllostomatidae), in which habitat diversity, roost types, and dietary opportunities (extrinsic factors) probably combined with developmental processes such as heterochrony and modified growth rates (intrinsic factors) to enable rapid diversification of skull shapes and sensory structures to exploit varied dietary niches.

The order of arrival might also play an important role in driving adaptive radiation through priority effects (Rosenzweig and McCord 1991, Reijenga *et al*. 2021, Foley *et al*. 2023, Stroud *et al*. 2024a). However, as mentioned above (‘Bridging the process–pattern divide’), such phenomena are often difficult to study in most adaptive radiations as these episodes would have occurred deep in the past (but see Ngoepe *et al*. 2023). Powell *et al*. (2024) provide an exception: in the radiation of cucujoid beetles, lineages that colonize a new resource first, so avoiding competition, tend to be the most diverse, thus providing indirect evidence for priority effects in this group.

### Genomic insights into adaptive radiation

Advances in genomic techniques are continuing to provide key insights into the genetic mechanisms contributing to adaptive radiation (Richards *et al*. 2021, Xiong *et al*. 2021, Marques *et al*. 2022, Patton *et al*. 2022, Richards and Martin 2022, Rubin *et al*. 2022, Cerca *et al*. 2023, Cicconardi *et al*. 2023, Meier *et al*. 2023, Wogan *et al*. 2023, Combrink *et al*. 2025), and the increasing ease and cost of generating genomic data means nonmodel groups can be investigated as outlined by papers in this special issue (e.g. Conith *et al*. 2024, Fenton *et al*. 2024, Layfield *et al*. 2024, Yang *et al*. 2024, Zapata *et al*. 2024).

Transposable elements (TEs) have been recognized as playing an important role in the generation of genetic diversity, but it remains unclear the extent to which they contribute to adaptive radiation. Yang *et al*. (2024) investigated how TEs accumulate in the Hawaiian *Tetragnatha* spiny-leg spider adaptive radiation, finding that these mobile genetic elements do not appear to be a key mechanism explaining their diversification. As most studies report positive roles for TEs in adaptive radiation, Yang *et al*.’s results may provide important evidence for a ‘file drawer’ bias (Rosenthal 1979) surrounding this topic (i.e. a publication bias towards positive results). Clearly, more adaptive radiations, non-adaptive radiations, and non-radiations need to be investigated to determine whether TEs are a major factor triggering adaptive radiations specifically. On a different oceanic island system, the Galápagos, Zapata *et al*. (2024) investigate the prickly pear cactus (*Opuntia* sp.) radiation using single nucleotide polymorphisms (SNPs), finding that despite extensive morphological diversity, genetic differentiation is limited, suggesting either that this radiation is in its early stages or that gene flow is ongoing. The finding of high morphological diversity coupled with shallow genomic differentiation and ongoing gene flow adds to the growing literature on young, isolated radiations, but which has typically been documented in fish systems (e.g. Ford *et al*. 2016, Martin and Feinstein 2014).

Increasingly in studies on adaptive radiation, diverse datasets (e.g. Grant and Grant 2024, Peart *et al*. 2024) are necessary to tease apart the mechanisms shaping adaptive radiations. Studies that combine genomic, phenotypic, and ecological data to investigate adaptive radiation are particularly powerful. By integrating environmental data in their study of Arctic charr (*Salvelinus alpinus*), a widely distributed fish species with sympatric ecotype populations, Fenton *et al*. (2024) demonstrate that the sizes of different lake ecosystems predict the potential for trophic specialization and the occurrence of sympatric divergent ecotypes, highlighting the role of ecological context in adaptive diversification.

This section on genomics has parallels to the first section (‘Bridging the process–pattern divide’) in highlighting a fundamental challenge of adaptive radiation research: while genomic methods have dramatically enhanced our ability to detect evolutionary patterns, inferring the underlying processes from these patterns remains complex and often ambiguous.

### Understudied groups and regions

While much of our understanding of ecomorphological diversification in adaptive radiations comes from well-studied vertebrate systems such as cichlid fishes (Fryer and Iles 1972) and *Anolis* lizards (Losos 2009), several studies in this special issue highlight important insights gained from traditionally understudied groups.

The African rift lakes, synonymous with the aforementioned cichlid radiations, also contain many other groups that provide fruitful avenues for understanding shared evolutionary processes and are brought into further focus in this issue. Peart *et al*. (2024) focus on two sympatric radiations of Lake Tanganyika catfishes (*Synodontis* and Claroteinae), which diversified over a similar time frame, but show differing degrees of ecological and morphological divergence. These authors find evidence for ecomorphological diversification and trophic niche partitioning in Claroteinae catfishes, whereas *Synodontis* shows more conserved patterns, suggesting different selective forces may have driven their diversification, or that intrinsic differences may limit selective responses in the *Synodontis* group. Similarly, van Bocxlaer *et al*. (2024) investigated the adaptive significance of shell morphology in two morphospecies of Lake Malawi gastropods (*Lanistes* sp.). Using a common garden experiment, van Bocxlaer *et al*. showed that differences in shell morphology linked to environmental variation across habitats were associated with differential fitness in the two taxa, suggesting shell characteristics may confer adaptive advantages in specific ecological contexts. The findings highlight how natural selection may shape phenotypic variation in gastropod populations, potentially driving speciation and ecological specialization. Further exploring the Lake Malawi system, Layfield *et al*. (2024) examine ecological speciation in freshwater crabs (Potamonatinae) across a lake–river boundary within the Lake Malawi catchment using genomic data. They show that divergent ecological pressures between lacustrine (lake) and riverine environments have led to the differentiation of populations, with distinct morphological and genetic traits emerging in response to habitat-specific factors, thereby driving speciation within this lineage.

In the radiation of San Salvador island pupfishes (*Cyprinodon* sp.) from the Bahamas, Chan *et al*. (2024) explore the relationship between phenotypic integration and morphological diversification by examining skull modularity across species and lab-reared hybrids. Despite extensive trophic specialization across species (Martin and Wainwright 2011), Chan *et al*. unexpectedly find a conserved modularity pattern, suggesting that the flexible trait associations within these modules, rather than among modules, probably underlie their rapid diversification.

Additional studies on understudied plant radiations reveal similar insights: dos Santos *et al*. (2024) explore niche evolution of Canary Island tree houseleek plants (*Aeonium* sp., Crassulaceae), showing distinct ecological niches have emerged in different island habitats driven by both allopatric and sympatric processes. By comparing species in isolated and overlapping environments, dos Santos *et al*. reveal how adaptive traits linked to resource availability, climate, and competition have shaped the evolutionary trajectories of this plant radiation. Similarly, as previously mentioned, Zapata *et al*. (2024) show extensive ecophenotypic variation in Galápagos prickly pear cacti (*Opuntia* sp.) despite evidence of limited genomic differentiation and high gene flow.

These contributions from understudied groups or regions expand our understanding of the diverse ways in which ecomorphological diversification can unfold in adaptive radiations. To establish general principles of adaptive radiation, it will be critical to continue studying obscure or overlooked groups. The reasons are twofold: both to broaden taxonomic sampling, and to uncover novel mechanisms and patterns that may remain hidden if research is constrained to historically well-studied groups.

### The important role of taxonomy and systematics

The importance of taxonomy and systematics in understanding adaptive radiations is critically examined in two complementary papers that challenge current practices and assumptions. Singhal *et al*. (2025) introduce a new concept of ‘taxon murk’—the multiple sources of uncertainty that affect species-level units used in phylogenetic analyses. The authors demonstrate this concept through extensive sampling of Australian lizards (sphenomorphine skinks), showing that diversification patterns can be robust despite taxonomic uncertainties. Specifically, an early burst pattern persists across different taxonomic frameworks (morphological, operational, incipient, and threshold), providing compelling evidence that some macroevolutionary signals transcend taxonomic ambiguity.

Turner (2024) addresses a different but related challenge, arguing that the rigid application of cladistic principles requiring species monophyly can lead to systematic underrepresentation of biodiversity in adaptive radiations. Using examples such as the Omani blind cavefish (*Garra barreimiae*) and Mexican cave tetras (*Astyanax mexicanus*), Turner (2024) demonstrates how current taxonomic practices often fail to recognize distinctive endemic forms because they would render widespread species paraphyletic.

Together, these papers suggest that while taxonomic uncertainty and practices can significantly impact our understanding of adaptive radiations, careful methodological approaches (as demonstrated by Singhal 2025) and more flexible taxonomic frameworks (as advocated by Turner 2024) can help resolve these challenges. Their work emphasizes that taxonomy should serve to illuminate rather than obscure evolutionary patterns, particularly in the context of adaptive radiations where novel phenotypes may evolve rapidly in geographically restricted populations.

## CONCLUSION

Our Editorial highlights two major takeaways. First, as also highlighted in another Editorial in this journal (Butlin 2024), making the connection between micro-scale process and macro-scale patterns remains a fundamental yet still challenging objective of evolutionary biology. This is especially true for increasing our understanding of the causes of adaptive radiation. One promising approach is the integration of ecology–morphology–performance–fitness data on adaptive landscapes and comparisons of these empirical distributions to theoretical adaptive landscapes; this integrative approach promises the best opportunity to understand how and why the observed form and function relationships evolved in a given adaptive radiation. Second, this special issue demonstrates the incredible value of combining diverse datasets and methods across diverse and sometimes obscure taxa. In bringing together broad perspectives spanning a range of organisms across the Tree of Life, the contributions in this issue not only advance our theoretical understanding of how biodiversity is generated and maintained but also point to promising emerging directions in the field.
